# Ophthalmoplegia in a Her2+ and β-hCG+ Patient With Leptomeningeal Carcinomatosis Secondary to Gastric Adenocarcinoma

**DOI:** 10.7759/cureus.26658

**Published:** 2022-07-08

**Authors:** Zachary Falk, Mei Bou Nasif, Nabil Fallouh

**Affiliations:** 1 Internal Medicine, George Washington University School of Medicine and Health Sciences, Washington, DC, USA; 2 Neurology, George Washington University School of Medicine and Health Sciences, Washington, DC, USA

**Keywords:** ophthalmoplegia, her2-positive, β-hcg, leptomeningeal carcinomatosis (lmc), gastric adenocarcinoma

## Abstract

Leptomeningeal carcinomatosis (LMC) is an uncommon and devastating late complication of metastatic malignancy that carries a poor prognosis, typically faring worse when secondary to solid tumors. Diagnosis of LMC can be challenging, especially if the underlying cancer is undiagnosed, as presenting symptoms can be nonspecific or involve focal deficits such as cranial nerve palsies. Typically, LMC can be recognized due to new central neurological findings with concomitant peripheral nerve involvement, but there has not been a case of LMC with isolated peripheral nerve findings to our knowledge. In this report, we present a case of LMC secondary to metastatic gastric adenocarcinoma in a patient whose only manifestation was cranial nerve palsies, and whose cancer was also found to be Her2+ and β-hCG positive, two markers not widely recognized in gastric cancer.

## Introduction

Leptomeningeal carcinomatosis (LMC) is defined as the dissemination of cancerous cells to the pia and arachnoid matter via the cerebrospinal fluid [1]. It is a rare complication that has been reported in solid tumors, more commonly lung cancer, breast cancer and melanoma [2], but occurs rarely in gastric cancer, at an incidence of 0.14%-0.24% [3-5]. It is a marker of poor prognosis with limited therapeutic options and a median survival of four weeks [1, 3]. Neurological symptoms are frequent in LMC and include a combination of central nervous system (CNS) and peripheral nervous system (PNS) symptoms. CNS symptoms include headache, confusion, seizures, weakness, ataxia, visual loss, behavioral and psychological changes. When the PNS (i.e., the peripheral portions of cranial nerves, dorsal root ganglia, peripheral portions of the spinal nerve roots, and peripheral nerves) is affected, symptoms can involve cranial nerve disturbances and can present as diplopia, ptosis, hearing loss, ageusia, anosmia, facial pain and facial numbness, as well as peripheral nerves, presenting with sensory or motor neuropathies. The most frequent symptoms in LMC are headache, dizziness, and confusion, and are usually accompanied by cranial nerve palsies [1]. We introduce a case of gastric signet ring adenocarcinoma that presented with new-onset isolated cranial nerve palsies secondary to LMC, in the absence of CNS symptoms.

## Case presentation

A woman in her fifties with a history of obesity, hypertension, type II diabetes mellitus, and recently diagnosed biopsy-confirmed localized gastric cancer presented to the emergency department with persistent nausea, vomiting, abdominal pain, and blurry vision for two weeks. One week prior to the presentation, she developed complete ptosis of her left eye. Her gastrointestinal symptoms were anorexia, fatigue, and unintentional weight loss of 50 pounds over the last two months. She denied diplopia, however, her family reported noticing her eyes crossing around three weeks prior to presentation. She denied vision loss, eye pain, photophobia, flashes, floaters, headache, dysphagia, tinnitus, hearing loss, facial pain, or numbness. Physical examination disclosed a hemodynamically stable and oriented woman in significant distress. Her neurological examination revealed ptosis, impaired adduction and elevation, and fixed 8-mm dilation of her left eye with a poorly reactive pupil (i.e., resting in the “down-and-out” position with the blown pupil when the eyelid raised), as well as impaired abduction of her right eye. The rest of her cranial nerves were normal on exam. Motor and sensory functions were intact except on her right thigh, where she demonstrated reduced strength on hip flexion due to pain, and she reported numbness on the right medial thigh as well. Otherwise, she did not display any signs of meningeal irritation and did not have any other focal neurological deficits. Of note, she had one small, nonmobile 1 cm x 2 cm nontender left supraclavicular lymph node palpated; no cervical lymphadenopathy was appreciated. An ophthalmologic exam showed good visual acuity, pressure, and a normal dilated fundus exam.

**Figure 1:**
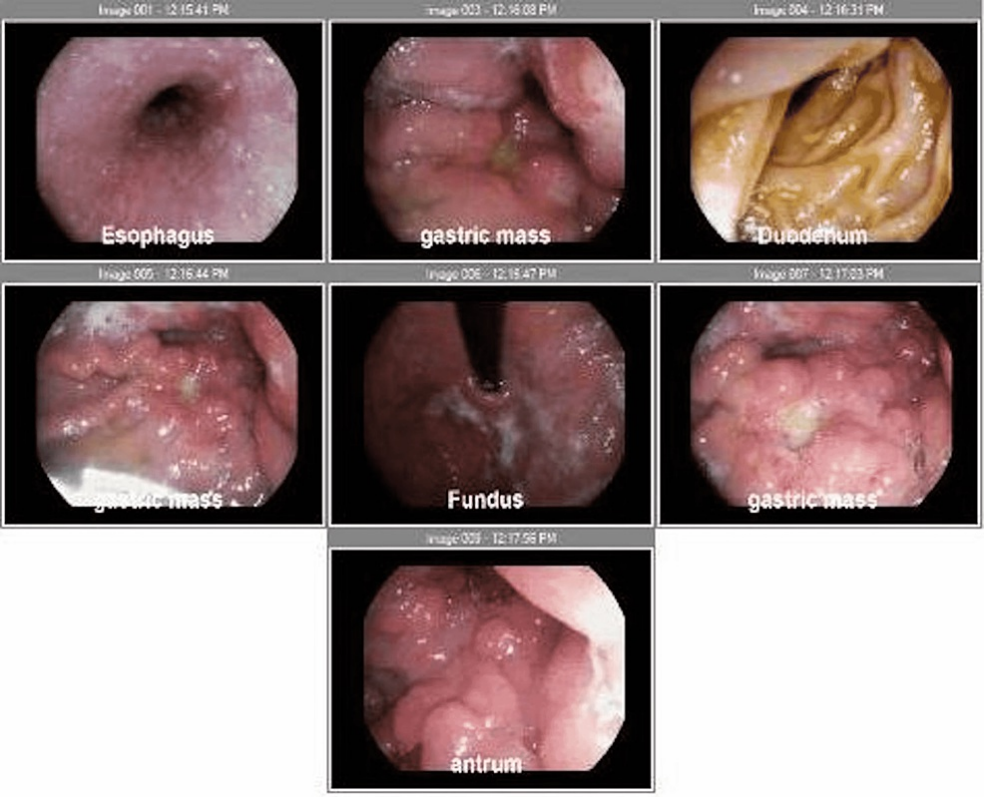
Gastric mass as seen from upper endoscopy.

Laboratory data was significant for mild leukocytosis, microcytic anemia, and mild transaminitis. The urine pregnancy test was positive, and subsequent beta-human chorionic gonadotropin (β-hCG) concentration was 118 IU/L, despite having undergone menopause five years prior. Recent gastric tumor biopsy via esophagogastroduodenoscopy (EGD) revealed poorly differentiated gastric carcinoma with focal signet ring features which was Her2-positive (score 3+), and fine needle biopsy of a celiac lymph node was positive for metastatic poorly differentiated carcinoma consistent with a gastric primary. The upper endoscopy images of the gastric mass are shown in Figure 1. 

**Figure 2:**
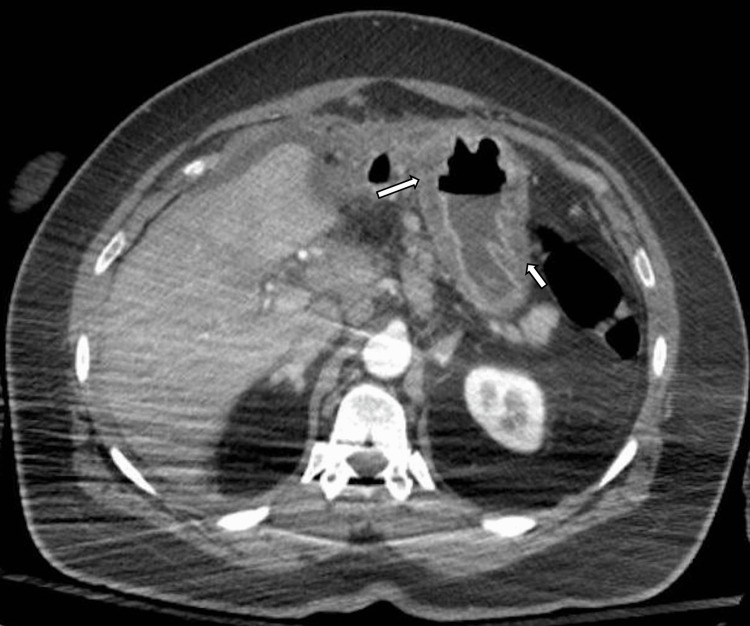
CT chest/abdomen/pelvis with contrast. CT chest/abdomen/pelvis with contrast demonstrating gastric wall thickening (arrows), perigastric stranding, edema, and free fluid.

CT was suggestive of gastric malignancy, peri-gastric and retroperitoneal adenopathy (Figure 2), and significant osseous metastatic disease including large lytic lesions in T11, L3, and L4 vertebral bodies and a 1.8-cm aggressive-appearing lytic lesion within the left occipital calvarium.

**Figure 3:**
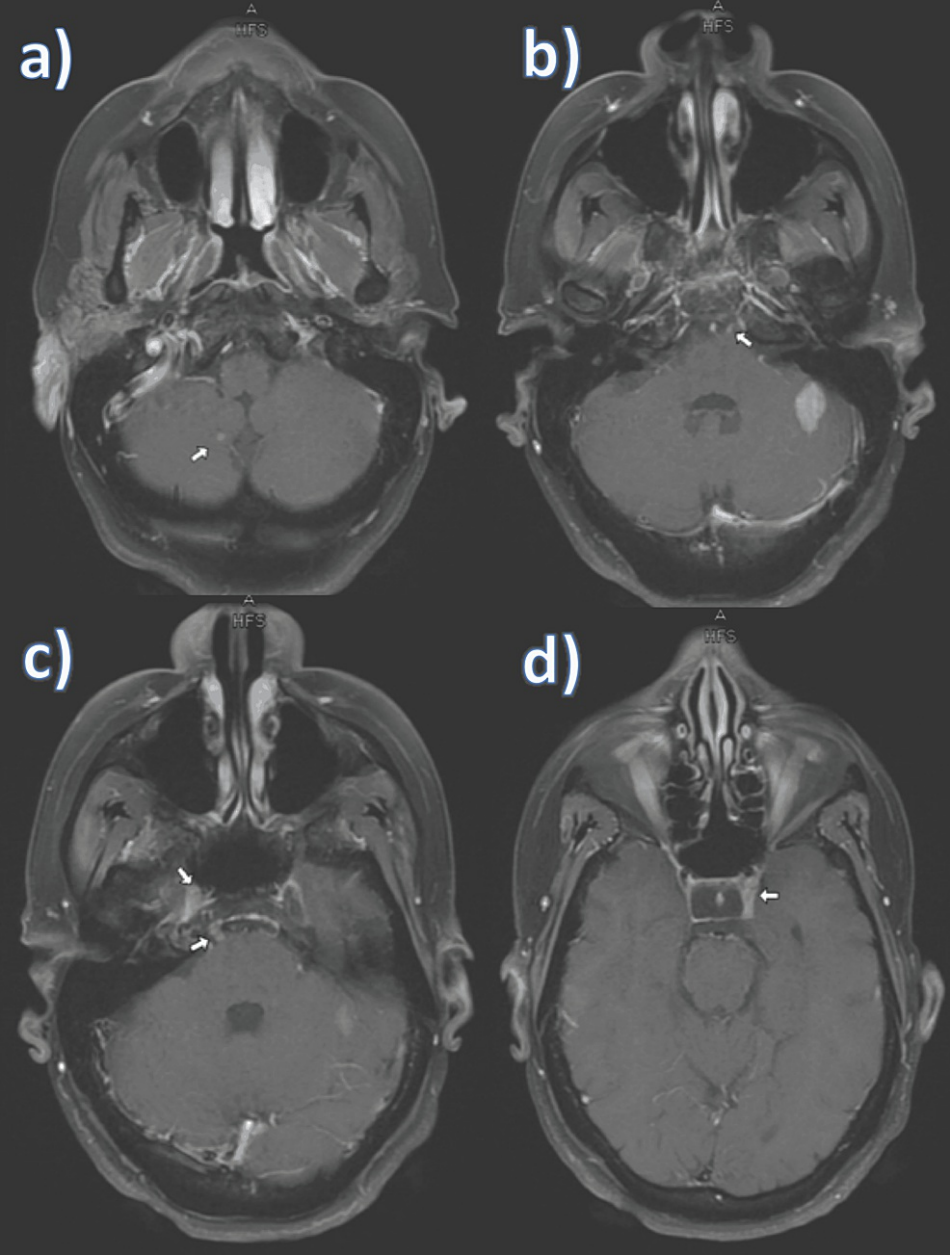
Brain MRI. a) Enhancing cerebellar lesion on the right. b) Abnormally-enhancing cerebellar lesion on the left and left cranial nerve (CN) VI enhancement. c) Enhancement of the right Meckel’s cave (upper arrow) and slight enhancement of the right CN VI. d) Bilateral enhancement of the prepontine cisterns.

The MRI of the brain showed asymmetric enhancement of the superior margin of the left cavernous sinus, right Meckel’s cave, and bilateral prepontine cistern with possible involvement of the left more than the right sixth cranial nerve with two abnormally enhancing cerebellar lesions (Figure 3). It also showed an enhancing left occipital bone lytic lesion and cerebrospinal fluid (CSF) seeding on the upper cervical spinal cord.

**Figure 4:**
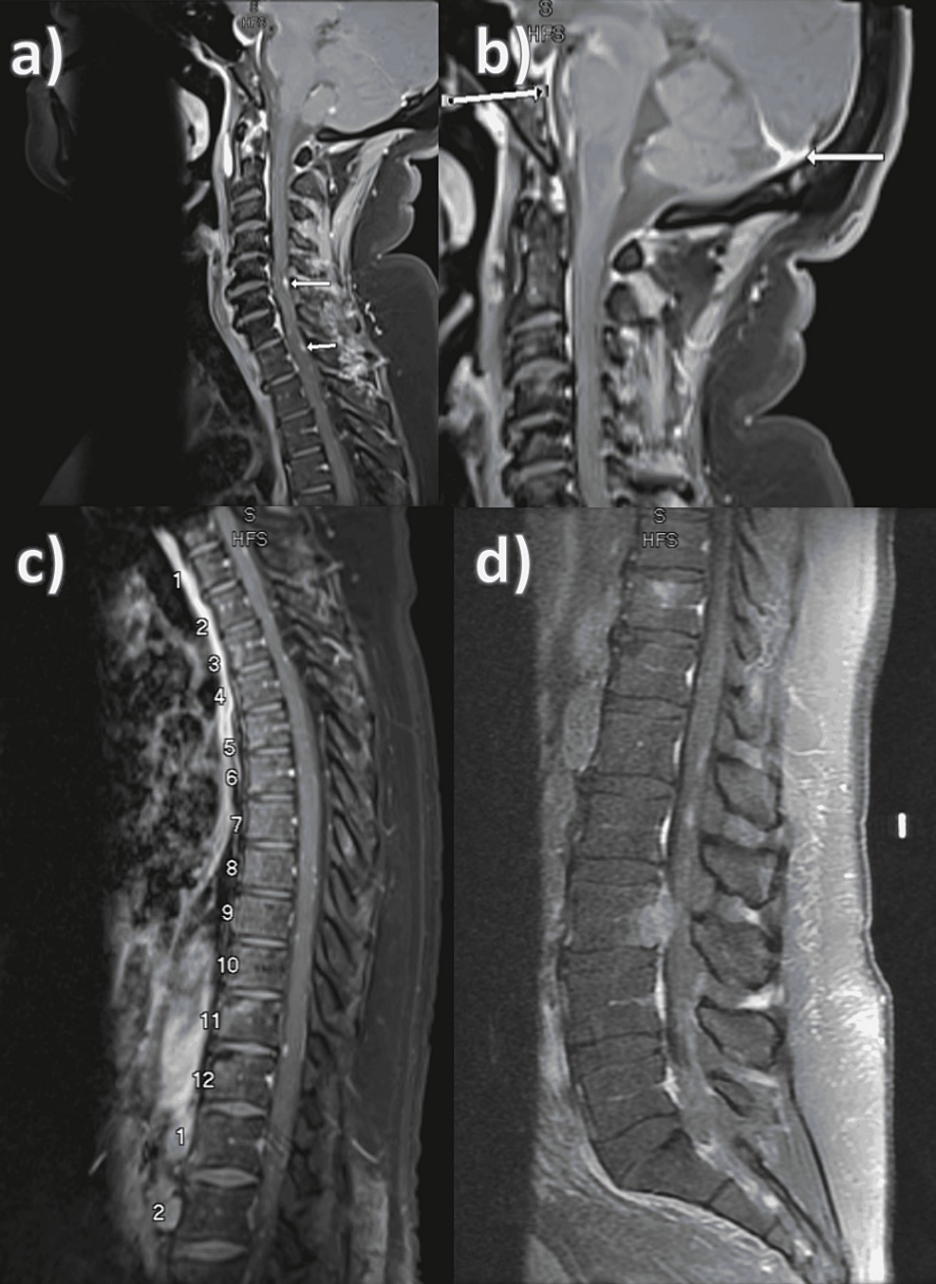
Spine MRI. a) Scattered leptomeningeal nodules along the cervical spine. b) Diffuse leptomeningeal enhancement. c) Scattered leptomeningeal nodules along the thoracic spine. d) Scattered leptomeningeal nodules along the lumbar spine.

Spine MRI disclosed multiple scattered punctate enhancing leptomeningeal nodules along the cervical spine. Left supraclavicular lymphadenopathy was also revealed, consistent with the nontender Virchow node appreciated on exam. Scattered leptomeningeal nodules were also seen along the thoracic spinal cord in addition to multiple intradural nodular enhancing foci involving the cauda equina and thecal sac narrowing at L3-L4 from the intraspinal extension of large metastases centered in the right pedicle of L3 (Figure 4). Lumbar puncture (LP) and CSF cytology investigations were not pursued given the high pretest probability of these lesions being malignant in nature secondary to gastric adenocarcinoma with carcinomatosis (T2N1M1 stage IV).

A gastrojejunal (GJ) tube was placed to circumvent the gastric outlet obstruction. She was recommended brain and spine radiotherapy and systemic chemotherapy with folinic acid, 5-fluorouracil, and oxaliplatin (FOLFOX), and trastuzumab. During her hospitalization, she received whole-brain radiotherapy and palliative spinal radiation without any changes in focal neurological symptoms. After completing her radiation therapy, she was deemed stable for discharge. She returned two days later with weakness, apparent gastrointestinal bleeding and respiratory distress, was intubated, and admitted to the intensive care unit (ICU). She was found to be bleeding from her gastric tumor and died from hemorrhagic shock. 

## Discussion

Leptomeningeal carcinomatosis is a rare complication that occurs in up to 5% of solid tumors. It has been most frequently associated with small cell lung cancer (9%-25%), breast cancer (2.5%-5%), and melanoma (23%) [2]. The pathophysiology of spread is variable; malignant tumor cells can disseminate to subarachnoid spaces by lymphatic, perineural, perivascular or hematogenous routes, as well as direct invasion from brain parenchyma or bone metastases in the calvarium or spine [6]. LMC secondary to gastric cancer is rarer, with only 0.14%-0.24% of gastric cancer resulting in leptomeningeal infiltration [1, 7]. It is most often associated with poorly differentiated adenocarcinoma with signet ring cell features [8], with studies suggesting that the predominant route taken by tumor cells is the Batson’s venous plexus to spread into the subarachnoid space [5]. LMC mostly affects the CNS and presents most frequently with headache, followed by visual loss [1]. Other ocular manifestations include diplopia, ptosis, papilledema, anisocoria, exophthalmos, orbital pain, scotoma, hemianopsia, and nystagmus [9]. A study by Oh et al. investigated the most common symptoms of LMC in 54 patients with gastric LMC and found that headache was the most frequent (85.1%), followed by nausea and vomiting (32%), dizziness (13%) and mental change (12%), while peripheral nerve involvement such as diplopia and ptosis were only reported in 5.6% and 1.9% of cases, respectively [7]. Cranial neuropathies have been associated with LMC to a lesser frequency than other neurological symptoms. There is a scarcity of reports describing cranial nerve palsies secondary to gastric cancer related LMC. A study by Lee et al. evaluated the neurological symptoms seen in 19 cases of LMC secondary to gastric cancer and found that the most frequently reported symptoms were headache, altered mental status, and that the oculomotor nerve and the abducens nerve were only affected in 13.3% of cases each [5]. While peripheral nerve involvement (such as cranial neuropathies) associated with LMC have been reported in the literature, they have been consistently described in combination with CNS findings such as headache, general weakness, seizures, vertigo, paraparesis, dysarthria, loss of consciousness, or confusion [8-15]. To our knowledge, PNS symptoms have not been described as an isolated finding when caused by LMC. Here we present an unusual case of LMC occurring in gastric cancer with only cranial nerve III and VI palsies, in the absence of CNS symptoms. Therefore, a high index of clinical suspicion of gastric adenocarcinoma is required despite subtle or seemingly unrelated symptoms, especially if a history of cancer is initially unknown to the clinician. Diagnosis usually involves gadolinium-enhanced brain and spine MRI as well as LP with CSF studies. MRI classically shows leptomeningeal enhancement and is helpful to perform prior to LP, as the LP can induce dural enhancement artifact, complicating interpretation. The gold standard for diagnosing LMC hinges on the presence of malignant cells seen in the CSF, but sensitivity is limited to approximately 55% on the initial LP investigation, yielding many possible false-negative results; a second LP can increase sensitivity to 85% [16-17].

Given that CSF cytology is not always technically feasible, MRI studies are often the initial and sole diagnostic tool for LMC. LMC has a harrowing prognosis, carrying a median survival time of two to four months for patients who undergo appropriate treatment [6, 18]. Further, it is well documented that LMC from a solid primary tumor source imparts a significantly worse prognosis compared to that which is derived from a hematological malignancy [19]. The fact that patients with LMC from a solid tumor source fare significantly worse than those with a primary hematologic malignancy are somewhat counterintuitive. It is generally accepted that some of this difference may be attributed to the later stage of diagnosis in solid tumors compared to their hematologic counterparts (median time to diagnosis is nearly double for solid tumor LMC) [3]. However, solid tumors have a propensity to adhere to neural structures and form nodules, which become visible on MRI. In fact, MRI with gadolinium sensitivity and specificity for LMC is greater for solid tumors than hematological malignancies [3]. Molecular changes such as matrix metalloproteinase, urokinase, and vascular endothelial growth factor (VEGF) upregulation have been hypothesized to play a role in pathogenesis, but these mechanisms are not necessarily specific to solid tumors.

The β-hCG has been most widely recognized as a marker of gestational trophoblastic neoplasms (e.g., choriocarcinoma) and germ cell tumors with trophoblastic elements (e.g., seminoma, embryonal carcinoma, teratoma). Up to 50% of nonseminiferous testicular tumors are found to secrete β-hCG, while the percentage of nontrophoblastic tumors overall is considerably lower [20]. A study by Murhekar et al. found that β-hCG was secreted in 18.7% of patients with gastric carcinoma [21]. In many cancers, the presence of β-hCG in serum is associated with poor prognosis, usually owing to its late-stage at detection, and is also related to chemoresistance, radioresistance, and increased invasiveness [22-23]. In oral squamous cell carcinoma, the expression of β-hCG was found to increase with the degree of dysplasia to malignancy in epithelia, lending support to the idea that the protein is involved in early carcinogenesis in some tumors [24]. In colorectal cancer, β-hCG may modulate the expression of epithelial‐to‐mesenchymal transition (EMT)‐related genes, including suppressing E‐cadherin, so it stands to reason that a similar mechanism may occur in gastric cancers [4]. However, a large immunohistochemical (IHC) study in India reported that the production of β-hCG was neither associated with the pathological grade nor the surgical stage in their large population of patients with gastric cancer [21]. A study by Zhao et al. found that measurable β-hCG in serum is rare compared to confirmed positive IHC staining for β-hCG [25]. This tissue-serum discrepancy makes it poorly suitable as a serum tumor marker for gastric cancer. Interestingly, our patient was also found to be Her2+ (score 3+) on a biopsy of her primary gastric mass. Much is still being learned about the prevalence and role of Her2 in gastric cancer. The reported frequency of Her2 overexpression in gastric cancer ranges from 4.4% to 53.4%, due in part to immunohistochemical/FISH discrepancies from heterogeneity in basolateral membranous immunoreactivity of glandular cells [26-27]. Her2 overexpression is generally thought to portend a poor prognosis and its overexpression seemingly differs on the basis of histologic subtype (intestinal vs diffuse), differentiation, and tumor location [28-29]. Others report that Her2 positivity is not independently prognostic of patient outcomes in metastatic gastric cancer, however [30]. A recent immunohistochemical study of 505 patients concluded that Her2 might be an appropriate biomarker for prognosis prediction as it was strongly related to disease recurrence, lymphatic invasion, and vascular invasion in stage I (IA and IB) gastric cancers [31]. Trastuzumab has been shown to have a meaningful survival benefit for this subset of patients, as demonstrated by the ToGA trial [32]. The combination of lapatinib, trastuzumab, and capecitabine has been shown in a case report to offer symptomatic relief and extend survival for LMC from Her2+ gastric cancer, although achieving therapeutic concentrations of these medications in CSF can be challenging [33]. Further investigation on this topic is warranted.

## Conclusions

Malignancy should always be explored and ruled out in a patient presenting with new-onset ophthalmoplegia. β-hCG remains underemphasized in the setting of metastatic gastric cancer, as it is more classically and commonly associated with choriocarcinoma and embryonal germ cell tumors. Her2 positivity may denote a more aggressive clinical course in gastric cancer; however, trastuzumab has made great progress in treatment. LMC, especially when derived from a solid tumor source, portends a grave prognosis, as shown by the rapid clinic decompensation seen in our patient.
